# Supplementary material for: Cell division machinery drives cell-specific gene activation during differentiation in Bacillus subtilis
Source: Proc Natl Acad Sci U S A. 2024 Mar 19;121(13):e2400584121. doi: 10.1073/pnas.2400584121 (PMC10990147; doi:10.1073/pnas.2400584121)
Supplement: Supplementary file 1 — Appendix 01 (PDF) [file pnas.2400584121.sapp.pdf]

PNAS

Supporting Information for

Cell division machinery drives cell-specific gene activation during differentiation in *Bacillus subtilis*

Sylvia Chareyre[a], Xuesong Li[b,c], Brandon R. Anjuwon-Foster[a], Taylor B. Updegrove[a], Sarah Clifford[a], Anna Brogan[a], Yijun Su[b,c], Lixia Zhang[d], Jiji Chen[d], Hari Shroff[b,c], and Kumaran S. Ramamurthi[a,1]

*Corresponding author: Kumaran S. Ramamurthi
Email: ramamurthiks@mail.nih.gov

This PDF file includes:

Figure S1
Table S1

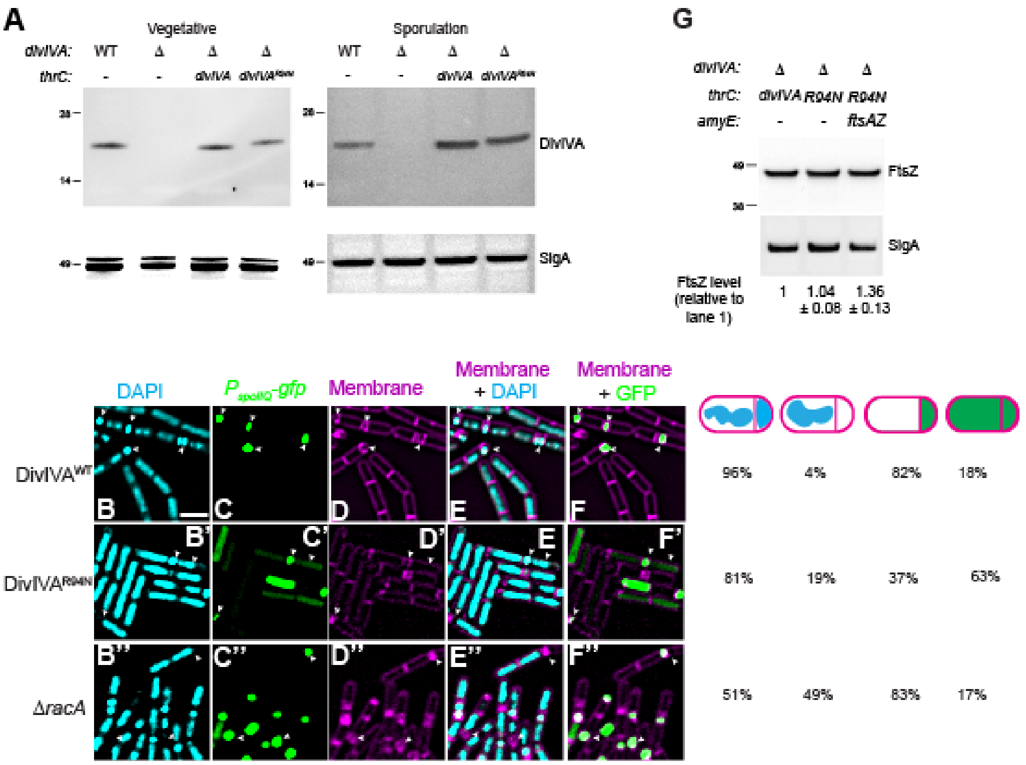

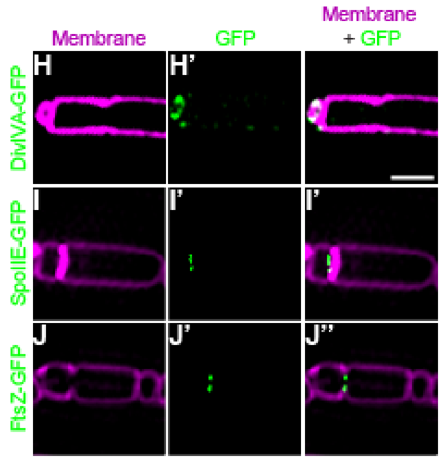

Figure S1. Characterization of DivIVA^{R94N}. (A) Immunoblot analyses of cell extracts of *B. subtilis* harvested during (left) vegetative growth or (right) 1.5 h after induction of sporulation using antiserum raised against purified DivIVA. Lane 1: WT; lane 2: Δ*divIVA*; lanes 3-4: Δ*divIVA* complemented with either *divIVA* or *divIVA*^{R94N}, respectively, at an ectopic chromosomal locus. Bottom panels: immunoblot using antisera raised against purified SigA used as a loading control. Strains: PY79, KR645, BRAF22 and APB8. (B-F'') Fluorescence micrographs of sporulating cells of *B. subtilis* producing (B-F) WT DivIVA, (B'-F') DivIVA^{R94N}, or (B''-F'') harboring a deletion of *racA* imaged 1.5 h after the onset of sporulation. (B-B'') chromosomes visualized using DAPI; (C-C'') fluorescence from GFP; (D-D'') membranes visualized using FM4-64; (E-E'') overlay, membranes and chromosome; (F-F'') overlay, membrane and GFP. (G) Representative immunoblot analyses of cell extracts of *B. subtilis* harboring (lane 1) WT *divIVA*, (lane 2) *divIVA*^{R94N}, or (lane 3) *divIVA*^{R94N} and an additional copy of *ftsAZ* at an ectopic chromosomal locus harvested 1.5 h after induction of sporulation using antisera raised against purified FtsZ. Quantification of FtsZ levels relative to WT (lane 1) from 3 independent cultures are reported below. Errors are S.D. (H-J'') Subcellular localization of (H-H'') DivIVA-GFP, (I-I'') SpoIIE-GFP, or (J-J'') FtsZ-GFP in vegetatively growing cells (monitored using dual-color 3D SIM) harboring a second copy of ftsAZ at an ectopic chromosomal locus that have been artificially induced to produce a polar septum by inducing SpoIIE production, imaged 2 h after incubation in rich medium. (H-J) Membranes visualized using FM4-64; (H'-J') fluorescence from GFP; (H''-J'') overlay, membrane and GFP. Scale bar: 2 μm. Strains SC786, SC791, SC792.

Table S1. *Bacillus subtilis, Escherichia coli* strains, and plasmids used in this study.

Strains

Name	Genotype	Source
Bacillus subtilis		
PY79	Prototrophic derivative of *B. subtilis* 168	(1)
KR546	*ΔdivIVA::erm*	(2)
PE118	*spoIIE-GFP-kan*	(3)
BRAF22	*ΔdivIVA::erm thrC::divIVA spec*	This study
APB8	*ΔdivIVA::erm thrC::divIVA*^{R94N} *spec*	This study
SJC124	*ΔdivIVA::cm thrC::divIVA spec sacA::P*$_{spoIIQ}$*-GFP kan*	This study
SJC93	*ΔdivIVA::cm thrC::divIVA*^{R94N} *spec sacA::P*$_{spoIIQ}$*-GFP kan*	This study
SC634	*ΔdivIVA::erm ΔspoIID ΔspoIIM thrC::divIVA spec amyE::divIVA GFP cat*	This study
SC635	*ΔdivIVA::erm ΔspoIID ΔspoIIM thrC::divIVA*^{R94N} *spec amyE::divIVA*^{R94N}*-GFP cat*	This study
SC656	*ΔdivIVA::erm ΔspoIID ΔspoIIM ΔspoIIQ thrC::divIVA spec spoIIE-GFP kan*	This study
SC657	*ΔdivIVA::erm ΔspoIID ΔspoIIM ΔspoIIQ thrC::divIVA*^{R94N} *spec spoIIE-GFP kan*	This study
SJC112	*ΔdivIVA::erm thrC::divIVA*^{R94N} *spec amyE::P*$_{ftsA}$*-ftsAZ cat*	This study
SJC125	*ΔdivIVA::erm thrC::divIVA*^{R94N} *spec sacA::P*$_{spoIIQ}$*-GFP kan amyE::P*$_{ftsA}$*-ftsAZ cat*	This study
SC527	*ΔdivIVA::erm thrC::divIVA*^{R94N} *spec amyE::P*$_{ftsA}$*-ftsA cat*	This study
SC529	*ΔdivIVA::erm thrC::divIVA*^{R94N} *spec sacA::P*$_{spoIIQ}$*-GFP kan amyE::P*$_{ftsA}$*-ftsA cat*	This study
SC544	*ΔdivIVA::erm thrC::divIVA*^{R94N} *spec amyE::P*$_{ftsA}$*-ftsZ cat*	This study
SC546	*ΔdivIVA::erm thrC::divIVA*^{R94N} *spec sacA::P*$_{spoIIQ}$*-GFP kan amyE::P*$_{ftsA}$*-ftsZ cat*	This study
SC688	*ΔdivIVA::erm ΔspoIID ΔspoIIM thrC::divIVA*^{R94N} *spec amyE::divIVA*^{R94N}*-GFP cat sacA::ftsAZ cat::tet*	This study
SC671	*ΔdivIVA::erm ΔspoIID ΔspoIIM thrC::divIVA*^{R94N} *spec spoIIE-GFP kan sacA::ftsAZ cat::tet*	This study
SC723	*amyE::divIVA-FLAG cat spoIIE-GFP kan divIVA::erm*	This study
SC724	*amyE::divIVA*^{R94N}*-FLAG cat spoIIE-GFP-kan divIVA::erm*	This study
SC786	*sacA::ftsAZ-cat::tet amyE::P*$_{hyperspank}$*-spoIIE-GFP spec*	This study
SC791	*sacA::ftsAZ-cat::tet zac86::P*$_{hyperspank}$*-spoIIE spec ftsAZ::ftsAZ-GFP erm*	This study
SC792	*sacA::ftsAZ-cat::tet zac86::P*$_{hyperspank}$*-spoIIE spec amyE::divIVA-GFP cat*	This study

SC800	*mNeonGreen-ftsA ΔspoIIM ΔspoIID thrC::divIVA spec divIVA::erm*	This study
SC801	*mNeonGreen-ftsA ΔspoIIM ΔspoIID thrC::divIVA^{R94N} spec divIVA::erm*	This study
SC807	*mNeonGreen-ftsA ΔspoIIM ΔspoIID thrC::divIVA^{R94N} spec divIVA::erm ftsAZ::cat::tet*	This study
SC802	*mNeonGreen-ftsZ ΔspoIIM ΔspoIID thrC::divIVA spec divIVA::erm*	This study
SC803	*mNeonGreen-ftsZ ΔspoIIM ΔspoIID thrC::divIVA^{R94N} spec divIVA::erm*	This study
SC809	*mNeonGreen-ftsZ ΔspoIIM ΔspoIID thrC::divIVA^{R94N} spec divIVA::erm ftsAZ::cat::tet*	This study
SC810	*ΔspoIIM ΔspoIID thrC::divIVA spec amyE::P$_{xyl}$-GFP-minD cat divIVA::erm*	This study
SC811	*ΔspoIIM ΔspoIID thrC:: divIVA^{R94N} spec amyE::P$_{xyl}$-GFP-minD cat divIVA::erm*	This study
SC831	*ΔspoIIM ΔspoIID thrC:: divIVA spec minCD::cat divIVA::erm thrC::divIVA spec*	This study
SC832	*ΔspoIIM ΔspoIID thrC:: divIVA spec minCD::cat divIVA::erm thrC:: divIVA^{R94N} spec*	This study
SC834	*ΔspoIIM ΔspoIID thrC:: divIVA spec minCD::cat divIVA::erm thrC:: divIVA^{R94N} spec sacA::ftsAZ::tet*	This study
Escherichia coli		
BTH101	*E.coli F' , cya-99, araD139, galE15, galK16, rpsL1 (StrR), hsdR2, mcrA1, mcrB1, relA1* Euromedex (ref. EUK001)	(4)

Plasmids

Name	Description	source
pKNT25	Derived from pSU40 Plac–MCS(HindIII–SphI–PstI–XbaI–BamHI–SmaI–KpnI–SacI–EcoRI)–T25	(4)
pUT18	Derived from pUC19. Plac–MCS(HindIII–SphI–PstI–SalI–XbaI–BamHI–SmaI–KpnI–SacI–EcoRI)–T18	(4)
pSC0209	SpoIIE-T18 derived from pUT18	This study
pSC0207	DivIVA^{WT}-T18 derived from pUT18	This study
pSC0210	DivIVA^{WT}-T25 derived from pKNT25	This study
pSC0211	DivIVA^{R94N}-T25 derived from pKNT25	This study

1. P. Youngman, J. B. Perkins, R. Losick, Construction of a cloning site near one end of Tn917 into which foreign DNA may be inserted without affecting transposition in Bacillus subtilis or expression of the transposon-borne erm gene. *Plasmid* **12**, 1-9 (1984).
2. P. Eswaramoorthy *et al.*, Cellular architecture mediates DivIVA ultrastructure and regulates min activity in Bacillus subtilis. *mBio* **2** (2011).

3. P. Eswaramoorthy, P. W. Winter, P. Wawrzusin, A. G. York, H. Shroff, K. S. Ramamurthi, Asymmetric division and differential gene expression during a bacterial developmental program requires DivIVA. *PLoS Genet* **10**, e1004526 (2014).
4. A. Battesti, E. Bouveret, The bacterial two-hybrid system based on adenylate cyclase reconstitution in Escherichia coli. *Methods* **58**, 325-334 (2012).
